# Peptide Toxins as Biothreats and the Potential for AI Systems to Enhance Biosecurity

**DOI:** 10.3389/fbioe.2022.860390

**Published:** 2022-03-08

**Authors:** Ying-Chiang J. Lee, Alexis Cowan, Amari Tankard

**Affiliations:** Department of Molecular Biology, Princeton University, Princeton, NJ, United States

**Keywords:** peptide toxins, biological weapons, biosecurity, synthesis, artificial intelligence, conotoxin, enterotoxin

## Abstract

Biological weapons have been used for thousands of years, but recent advances in synthesis technologies have made peptide and protein toxin production more accessible and pose a threat to biosecurity worldwide. Natural toxins such as conotoxins, certain hemolytic compounds, and enterotoxins are peptide agents that can be synthesized in an environment with weak biosecurity measures and rudimentarily weaponized for limited use against smaller targets for lethal or nonlethal effects. Technological advances are changing the threat landscape around biological weapons and potentially facilitating a shift from state sponsored to more micro-level threats stemming from terror cells, insider threats, and lone wolf attacks. Here, we present the reader with an overview of the threat of peptide and protein toxins, provide examples of potent peptide toxins, and introduce capabilities of a proposed biosecurity program utilizing artificial intelligence that unifies commercial nucleotide and peptide synthesis vendors.

## Introduction

The use of biological agents and toxins as biological weapons (BW) have been documented throughout history, with some of the earliest uses involving Scythians dipping their arrows in decomposing cadavers of adders and human blood hypothesized to have contained *Clostridium perfringen*s and *Clostridium tetani* (Barras and Greub, 2014). The use of toxin tipped darts and arrows have been documented from ancient India to South America (Bisset and Mazars, 1984; Nedergaard, 2003). By the end of the 19th century, scientific advances in microbiology would form a knowledge base and pave the way for the early state-level BW programs established during World War I and beyond.

The threat of biological warfare combined with the devastation caused using chemical weapons during WWI led to the Geneva Protocol of 1925 that prohibited the use of gas and bacteriological methods of warfare. However, this protocol did not prohibit the research or production of biological weapons, and several countries started BW research and development. Imperial Japan’s BW program was made infamous by Unit 731 where it studied and used pathogens such as plague, cholera, and typhoid, on Chinese prisoners and cities (Guillemin, 2006; Wilson and Mari, 2019). In 1972, the Biological and Toxin Weapons Convention (BWC) outlawed the development, production, stockpiling, and acquisition of BWs for offensive purposes indefinitely (Grönvall, 2005). The BWC has 183 state-parties, but several signatories have committed violations. The Biopreperat in the USSR, established after the BWC, employed 60,000 people, and developed BW capabilities under the cover of legitimate biotechnological and pharmaceutical research (Davis, 1999; Grönvall, 2005). Several other countries such as Iraq and South Africa with its infamous Project Coast are also known to have researched, developed or actively used biological agents including toxins (Leitenberg, 2001; Roffey et al., 2002; Bale, 2006). In addition to the BWC, toxins are also regulated by the Chemical Weapons Convention.

BW research, development, and use have not only been limited to nation-states. Often referred to as the poor man’s nuclear bomb, individuals, religious cults, and terror organizations have taken interest in, procured, and in several cases, deployed biological agents (Carns, 2000; Carus, 2001; Salama and Hansell, 2005)^1^. While these cases generally involved rudimentary BWs and methods, the ever-expanding bioeconomy coupled with an increasing number of graduates studying biological sciences around the world are setting the scene for a future use of BWs, not only from state actors, but also non-state actors and individuals. Terror networks and their cells may also have the capacity and capability to engage in research of BWs as seen with Al Qaeda in Afghanistan (Salama and Hansell, 2005).

While pathogenic organisms, proteinaceous toxins, and small molecule toxins have garnered significant attention as BW agents, peptide toxins present a unique and perhaps underappreciated threat as noninfectious, synthesizable weapons. Both proteinaceous and small molecule toxins currently require extraction and purification from natural or heterologous expression sources; however, peptide toxins can also be chemically synthesized. Significant advances in biotechnology and current loopholes in nucleic acid and peptide synthesis security highlight a changing threat landscape. In this article we will explain the threat of peptide toxins, provide some examples of naturally occurring peptide toxins, and present a proposed, conceptual framework for an artificial intelligence network to increase biosecurity for commercial nucleic acid and peptide synthesis providers.

## The Threat of Peptide Toxins

Currently, the United States Centers for Disease Control categorizes biological agents and diseases of concern based on availability, ease of production and dissemination, ability to cause social disruption, the potential for high levels of morbidity and mortality, and special actions for public health preparedness (Rotz et al., 2002). Toxins are integrated into the list as products and effectors of pathogens. While toxins are often thought of along with their microbial producers, the threat from toxins as a separate entity has been seen before - notably with ricin toxin where 210-350 ug can be lethal to a 70 kg adult if inhaled or injected (Moshiri, Hamid, and Etemad, 2016). Toxins do not need an incubation period, and act directly on a target. They are also inherently self-limiting and without the capability to self-replicate like infectious BW agents.

Toxins are found as small molecules or peptides and proteins; however, the threat from peptide toxins is unique due to their small size compared to protein toxins and relative ease of and access to synthesis methods compared to small molecules. Protein-based toxins such as ricin, Staphylcoccal enterotoxin B, and botulinum toxin are too large to be synthesized by benchtop peptide synthesizers. Small molecule toxins such as saxitoxin, tetrodotoxin, and T-2 mycotoxin, require multiple biosynthetic steps. Peptide toxins can be synthesized through more straightforward biological and chemical methods. Biological synthesis in a laboratory setting can take advantage of not only natural producers but also through heterologous and recombinant protein expression. Peptide chemical synthesis involves solid state chemistry with equipment and reagents readily accessible to both commercial and academic laboratories but does have a limitation in that some sequences and structures can be difficult to properly synthesize. Both biologically and chemically synthesized peptide toxins can then be further modified, purified, and rudimentarily weaponized using conventional laboratory methods and equipment.

Peptide toxins originate from many diverse sources, from bacteria to marine organisms and plants. The bacterial exotoxin STa is a ribosomally synthesized, heat stable peptide 18 (STp) or 19 (STh) amino acid peptide secreted by enterotoxigenic *E. coli* (Weiglmeier, Rösch, and Berkner, 2010; H.; Wang et al., 2019)*.* STa binds to the guanylate cyclase C receptor on the brush border of intestinal epithelial cells, disrupting electrolyte balance, and causing diarrhea. STa has no reported median lethal dose (LD_50_) and diarrhea itself is not usually lethal for adults where there is access to clean water and basic medical services; however, diarrhea can nonlethally incapacitate a fighting force or disrupt civilian life as seen with the 1984 Rajneeshee plot to disrupt a local election (MacIntyre, 2015). The 15-residue linear peptide gramicidin is a mixture of isoforms and produced by the soil bacterium *B. brevis* (David et al., 2013)*.* Two gramicidin peptides orient end-to-end at target cell membranes forming an ionophore that depolarizes the membrane with an egress of ions from the cell eventually leading to cell death. While approved for topical antimicrobial use, gramicidin induces hemolysis at 0.5 ug and was shown to be cytotoxic at less than 1 uM (Herrell and Dorothy, 1941; David et al., 2013). Many marine organisms produce toxins for defensive measures or predation. One of the most well-known marine toxins, conotoxins, are known to target voltage gated channels to nicotinic acetylcholine receptors (Bjørn-Yoshimoto et al., 2020). More than 80,000 peptide conotoxins generally ranging from 10–35 amino acids are thought to exist among an estimated 700 species of cone snails with one species, *Conus geographus,* known to produce toxins lethal to humans at doses as low as 0.029 mg/kg (Dutertre et al., 2014; Gao et al., 2017; Safavi-Hemami et al., 2019). Fungi also produce powerful toxins that can have lethal or incapacitating effects. Among these are cyclic peptides: peptides whose C- and N- termini are linked together through an amide or other chemically stable linkage to form a ring-like structure (Joo, 2012). The amatoxins produced by mushrooms within the genera *Amanita*, *Galerina*, and *Lepiota* consist of nine distinct cyclic octapeptides that bind to and inhibit eukaryotic RNA polymerase II with high specificity and affinity (Göransson et al., 2012; Walton, 2018). Upon ingestion, amatoxins stably pass through the digestive system and enter the circulation rapidly. Amatoxins are responsible for the vast majority of fatal mushroom poisoning incidents, with LD_50_ values around 0.3 mg/kg. In addition to amatoxins, mushrooms in the genus *Amanita* also produce phallotoxins. These cyclic heptapeptide toxins bind with high affinity to actin, disrupting cytoskeletal dynamics and preventing muscular contraction. Phallotoxins possess an LD_50_ around 2 mg/kg, however ingestion of these toxins is not lethal as their structure destabilizes within the digestive tract (Walton et al., 2012; Walton, 2018). To exert their toxic effects, phallotoxins must be injected or absorbed through the skin (Walton et al., 2012).

The threat of peptide toxins should be assessed with an overview of the barriers and context that they exist in. We do need to acknowledge that production of a weaponized peptide at scale for use on a large population is neither simple nor easily attainable. Peptide toxins are not, by nature, optimized for spread. Unlike biological agents such as anthrax, plague, and smallpox, peptides are noninfectious and result in zero transmissibility when separated from their producers. Naturally occurring proteases and environments such as the low pH found in the stomach and serum are known to degrade proteins and shorten their half-life. The synthesis—either biological or chemical—of a peptide toxin at a scale able to cause mass casualties is also a limiting factor. However, the barriers to producing a quantity of toxin capable of affecting a single individual or group of individuals are many orders of magnitude lower. Production of a few grams that could be used to harm a small number of people does not present a major financial burden for most groups or even lone actors motivated to use a biochemical approach to causing harm. The biological or chemical production of the toxin could even be conducted in a research lab disguised as legitimate research, without awareness from others working in the same space. Thus, if a peptide toxin were to be used in a biocrime, it would most likely appear as a targeted poisoning of individuals or a group by someone trained in the biosciences rather than something used on a large scale by terrorists seeking broad harm.

## The Use of Artificial Intelligence in Proactive Biothreat Detection

Current deficiencies in regulations governing peptide synthesis are potential points of exploitation. Despite federal guidance for nucleic acid synthesis security in the United States and international consortia of gene synthesis providers that aim to promote best practices and biosecurity, there lacks a formal, enforceable, federal law on nucleic acid synthesis security screening (Hayden, 2009; Diggans and Leproust, 2019). Passing such a bill into law has not been simple. The governor of California recently vetoed a bill that would mandate scientists to purchase synthesized genes from companies that conduct both customer and sequence screening, citing the inefficiency of patchwork regulations at the state and federal level (West and Gronvall, 2020)^2^.

While attention has been largely focused on nucleic acid synthesis and DNA sequencing security, there are no formal recommendations at the federal level for peptide synthesis and attempts at formalizing peptide synthesis security may encounter similar resistance as seen for nucleic acid synthesis. A comprehensive approach that reaches all commercial vendors is needed to ensure that toxic peptides and the sequences that encode them are not synthesized.

**FIGURE 1:**
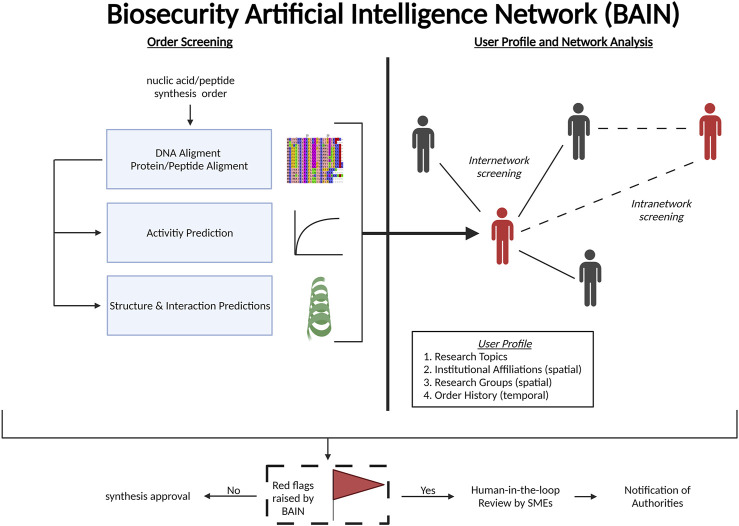
Framework of the biosecurity artificial intelligence network (BAIN).

Artificial intelligence (AI) presents as a powerful tool that can alert to potentially dangerous genetic and peptide constructs and can be leveraged extensively for biosecurity. AI has been proposed to be an asset in monitoring, managing, and responding to biothreat events (Su et al., 2021). For our purposes, a central AI that integrates across the entire commercial biological synthesis space can unify security and screening protocols among all nucleic acid and peptide synthesis providers in a screening-as-a-service (SaaServ) package. Here, we describe desirable critical capabilities of such a conceptual system that we refer to as a biosecurity AI network (BAIN) that could improve biosecurity in the biological research domain (Figure 1).

BAIN would screen every commercial nucleic acid order (including gene blocks and oligonucleotides) and peptide order against a database containing genomic and proteomic data of known pathogens as well as toxic peptides and proteins. This is similar to a United States Intelligence Advanced Research Projects Agency initiative launched in 2016 (Reardon, 2019). We also include nucleic acid synthesis security due to the possibility of obfuscation methods and the goal of a holistic approach to synthesis biosecurity. During the nucleic acid screening process, BAIN would also incorporate detection of primer binding sites of concern. While the current focus for screening is on synthesis of longer nucleic acid sequences, short oligonucleotides could be used as primers to clone peptide toxins and other genes of concern from an organism (of both pathogens and non-pathogens). In this case, the oligonucleotides themselves are initially seen as harmless, but the sequence that a pair of these primers flank may be extracted and encode a toxin or other harmful product. In addition, screening of oligonucleotides, and not just longer gene sequences, is also important due to the possibility that short peptide toxins could be encoded on the oligonucleotides themselves.

BAIN will also take advantage of machine learning (ML) programs for *in silico* bioactivity prediction that is integrated into screening procedures (Figure 1). *In silico* bioactivity prediction allows for potentially toxic or dangerous gene products to be identified and flagged for further inspection. In the past decade, ML programs have been created that take an input of amino acid sequences and predict antimicrobial, DNA-binding, ion channel inhibiting, inflammatory, and hemolytic activities (Ding et al., 2014; Wang et al., 2016; Blanco-Míguez et al., 2017; Timmons et al., 2020; Etzion-Fuchs et al., 2021)). Further iterations of BAIN with expanded computing resources may predict peptide and protein structures as well as potentially concerning peptide or protein interactions of all synthesis orders by integrating with existing programs such as RoseTTAFold and DeepMind’s AlphaFold2 (Baek et al., 2021; Jumper et al., 2021).

To fully utilize its AI capabilities, BAIN’s main innovation in the biosecurity space will be its ability to compile customer profiles, orders, screening results, and build a user network that also categorizes each user’s research areas using user-submitted data and web scraping tools (Figure 1). Information such as an individual’s affiliations and research group can be gathered to create a network map of researchers that would help BAIN detect abnormalities. BAIN would also pair primers as well as other nucleic acid and peptide synthesis orders from an institution or nodes from the research network map to identify purposeful obfuscation of potentially malicious nucleic acid or peptide synthesis orders. A bad actor may attempt to spread out orders temporally (by ordering sequences months apart) or spatially (by utilizing the synthesis order services of different coworkers or at different collaborating research facilities), and BAIN’s network map would be a step in countering this type of initially decentralized threat. All red flags raised by BAIN will be reviewed by subject matter experts that will then recommend further actions as deemed appropriate.

One point of consideration is use of the BAIN SaaServ. While we see no major hurdles from synthesis providers in joining BAIN—they may be encouraged to join as a way of advertising their dedication to biosecurity—but pharmaceutical and biotechnology companies may be hesitant due to the potential for network incursion and loss of intellectual property and/or patent rights with data centralization within BAIN. To prevent this, the BAIN SaaServ should be designed to be implemented within a company network, or even within a company division or geographical office as a SaaServ program and not link back to the central BAIN that exists. Thus, two versions of BAIN can exist—a main one and one sold to companies as a standalone program. This “company” version of BAIN would protect research interests by keeping all screening, profiling, and data storage within the company’s servers, and if outside synthesis vendors are used, screening would occur within the company itself prior to the order being released to the synthesis vendor. Red flags and SME evaluation would be handled internally. Periodic updates to BAIN’s screening checklist and capabilities can be introduced as software updates for companies wishing to use this standalone version of BAIN. We also expect university research groups to utilize the main BAIN without much difficulty—researchers often have profiles with synthesis vendors that maintain a list of all previous orders and consolidating such information within BAIN would not present as a major change from current practices. However, should academic organizations choose to use the standalone version of BAIN, they are also free to. This would decrease the internetwork screening capabilities of BAIN but use of BAIN’s SaaServ would still offer a high level of biosecurity within the organization.

The SaaServ framework for BAIN is diagrammed here. The same format would be used for the main version of BAIN as well as the standalone company version. BAIN would provide enhanced biosecurity measures for commercial vendors on an international scale. Nucleic acid and peptide synthesis orders will be screened by BAIN. User data will be mined and collected from open source, vendor information, and institutional resources to compile a user profile that automatically groups and links researchers. Red flags raised by BAIN will be forwarded to subject matter experts (SMEs) who review the information before deciding to notify the authorities. Created with (Figure 1).

## Conclusion

Biotechnological advances are changing the threat landscape that biological weapons and toxins present in the modern era. These advances have made diverse, naturally sourced peptide toxins more accessible to bad actors and highlights the need for formalized regulations and laws that govern nucleic acid and peptide synthesis orders. To that end, a holistic, biosecurity approach using artificial intelligence that combines multiple layers of screening and prediction with user network analysis in the BAIN package outlined in this article will help provide a level of biological arms control and counterproliferation that is needed. BAIN’s security net will ultimately rely on trust and sustained cooperation among all parties involved. However, BAIN has several loopholes. BAIN cannot screen synthesized products created without the aid of commercial synthesis vendors such as with portable synthesizers or though extraction and purification from natural or bioengineered sources. DNA synthesis orders could also use NNK codons combined with screening methods to isolate peptide toxin encoding sequences without raising alarms. BAIN also cannot detect sequences not submitted to its vendor network - nucleic acids sequences could be sequenced in most laboratories and peptide synthesizers could be used to create toxins outside the purview of BAIN. Finally, BAIN would utilize ML programs trained on databases which could be susceptible to adversarial poisoning. Despite these loopholes, BAIN serves as a conceptual framework for enhanced biosecurity among commercial synthesis vendors and users that is needed to counter biothreats before they are created and, at the least, serves as a deterrent.

## Data Availability

The original contributions presented in the study are included in the article/Supplementary Material, further inquiries can be directed to the corresponding author.

## Footnotes

1 Harald Doornbos and Jenan Moussa. Found: The Islamic State’s Terror Laptop of 38 Doom. *Foreign Policy* (2014).

2 Governor Newsom of California vetoes bill AB 70 on gene synthesis providers by Assemblymember Rudy Salas (D-Bakersfield). Published 05 October 2021.
